# Tracking the rising extinction risk of sharks and rays in the Northeast Atlantic Ocean and Mediterranean Sea

**DOI:** 10.1038/s41598-021-94632-4

**Published:** 2021-07-28

**Authors:** Rachel H. L. Walls, Nicholas K. Dulvy

**Affiliations:** Earth to Ocean Research Group, Department of Biological Sciences, Simon Fraser University, Burnaby, BC V5A 1S6 Canada

**Keywords:** Ecology, Ecology, Environmental sciences, Environmental social sciences, Ocean sciences

## Abstract

The loss of biodiversity is increasingly well understood on land, but trajectories of extinction risk remain largely unknown in the ocean. We present regional Red List Indices (RLIs) to track the extinction risk of 119 Northeast Atlantic and 72 Mediterranean shark and ray species primarily threatened by overfishing. We combine two IUCN workshop assessments from 2003/2005 and 2015 with a retrospective backcast assessment for 1980. We incorporate predicted categorisations for Data Deficient species from our previously published research. The percentage of threatened species rose from 1980 to 2015 from 29 to 41% (Northeast Atlantic) and 47 to 65% (Mediterranean Sea). There are as many threatened sharks and rays in Europe as there are threatened birds, but the threat level is nearly six times greater by percentage (41%, *n* = 56 of 136 vs. 7%, *n* = 56 of 792). The Northeast Atlantic RLI declined by 8% from 1980 to 2015, while the higher-risk Mediterranean RLI declined by 13%. Larger-bodied, shallow-distributed, slow-growing species and those with range boundaries within the region are more likely to have worsening status in the Northeast Atlantic. Conversely, long-established, severe threat levels obscure any potential relationships between species’ traits and the likelihood of worsening IUCN status in the Mediterranean Sea. These regional RLIs provide the first widespread evidence for increasing trends in regional shark and ray extinction risk and underscore that effective fisheries management is necessary to recover the ecosystem function of these predators.

## Introduction

Overfishing is the most imminent threat to many marine organisms^1^. Governments need repeated comprehensive assessments of extinction risk to effectively monitor the status of marine biodiversity^2^ and track progress towards global biodiversity and sustainable development targets^3^ if they are to halt declines, prevent more local extinctions, and recover species. The Red List Index (RLI) tracks the changing extinction risk of groups of species based on status changes recorded on the International Union for Conservation of Nature Red List of Threatened Species^4^ (hereafter ‘IUCN Red List’). The RLI includes species listed under six categories, in ascending order of threat: Least Concern, Near Threatened, Vulnerable, Endangered, Critically Endangered, and Extinct, but excludes the Data Deficient category^4^. Thus far, the RLI has mostly been applied to terrestrial species, including birds, mammals, amphibians, and cycads^5–8^. Our only understanding of changing marine extinction risk from the RLI so far comes from its application to stony corals, which reveals the threat of climate change to a key group of foundation species in the tropical oceans^9^. There remains a pressing need to develop an extinction risk trajectory representative of widely distributed marine fishes that are primarily threatened by overexploitation to better-understand the effects of this more immediate threat.

The RLI is eventually intended for global-level application, but like the Red List Assessments themselves, it is also informative when applied at regional and national scales^10^ or decomposed by biome, habitat, conservation profile, or international treaty^5^. Here we focus on a regional Red List assessment because these finer-scale analyses better-connect species status and trajectory to local and regional variation in threats and conservation management across socio-political and economic regimes^11,12^. We focus on the sharks, rays, and ghost sharks (Class Chondrichthyes, herein ‘sharks and rays’) in the Northeast Atlantic Ocean and Mediterranean Sea for four reasons: (1) sharks and rays have the greatest percentage of ‘threatened’ species (i.e. Vulnerable, Endangered, or Critically Endangered) of any taxonomic Class of marine organisms^13^, with numerous populations worldwide already locally or regionally extinct due to overfishing^1,14,15^, including many from the Northeast Atlantic and Mediterranean Sea^16–18^; (2) the status of sharks and rays in this region has been comprehensively assessed twice: in 2003/2005 and in 2015^19–21^; (3) the Northeast Atlantic and Mediterranean Sea may be a ‘canary-in-the-coal mine’ for collapse and recovery of fish populations because of the combination of a long history of exploitation^22^, high scientific capacity^17^, substantial modern fishing effort^23^, and divergent patterns of fisheries development and management^24^; and (4) extinction risk categorisations have already been predicted for the Data Deficient species in these regions in previous research^25^, presenting the opportunity to include these Data Deficient species in the RLI as well. One of the key challenges for the development of a taxonomically representative indicator is the high number of Data Deficient species listed, which until now have been excluded from trajectories of extinction risk even though some of them are predicted to be at risk^25^.

Here, we provide a regional synthesis of the changing extinction risk for sharks and rays in the Northeast Atlantic and Mediterranean Sea (the RLI). We retrospectively assign IUCN categorisations to each species for the year 1980 through a critical review of historical fishing patterns and scientific literature^4,7,26^. In our previously published research we predicted the IUCN categorisations of all Data Deficient species in 2015 (*n* = 21 Northeast Atlantic, *n* = 12 Mediterranean)^25^, which we now incorporate into the RLIs alongside the IUCN-assessed categorisations (*n* = 98 + 21 = 119 Northeast Atlantic, *n* = 60 + 12 = 72 Mediterranean). We disaggregate the RLIs by primary habitat, then further explore the biological and ecological traits that are related to extinction risk. We build on the knowledge that sharks and rays with slower life histories (i.e. slower growth and population turnover rates^27–29^) and shallower depth distributions (i.e. higher exposure to fishing activity due to limited depth refuge^30^) are more likely to be categorised as threatened on the IUCN Red List^25,30^ by exploring the likelihood of shark and ray Red List status worsening between assessment years based on these biological and ecological traits.

## Results and discussion

### Changes in extinction risk from 1980 to 2015

We compiled the results from the first (2003 Mediterranean Sea^19^, 2005 Northeast Atlantic^20^) and second (2015^18,21^) regional IUCN Red List assessments of sharks and rays, then ‘backcast’ (i.e., retrospectively assigned an IUCN status) to 1980 through a critical review of historical fishing patterns and scientific literature^4,7,26^ (see Methods for further detail and Supplementary Information for species-specific backcasting justifications).

**Figure 1:**
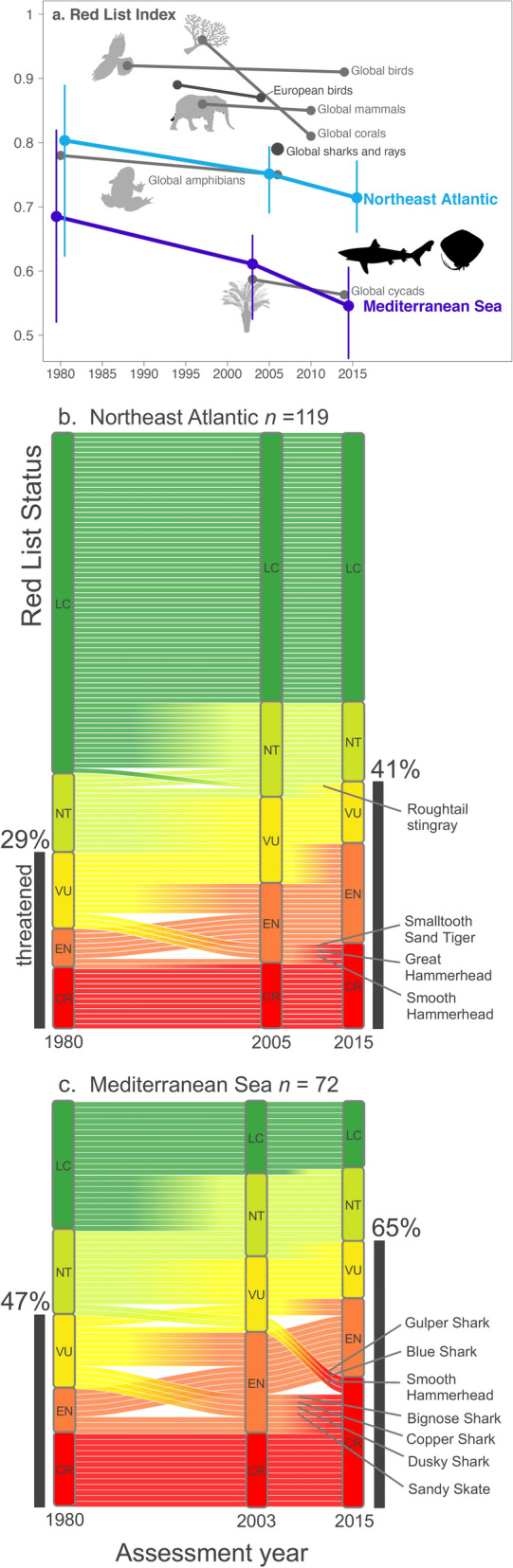
Worsening status of Northeast Atlantic Ocean and Mediterranean Sea sharks and rays from 1980 to 2015. (**a**) Regional RLIs for sharks and rays in the Northeast Atlantic (*n* = 119; blue line) and Mediterranean Sea (*n* = 72; purple line), for birds in Europe (*n* = 522^34^), and global RLIs for hard corals (*n* = 704^9^), birds (*n* =  ~ 9839^6^), mammals (*n* = 4653^7^), amphibians (*n* =  ~ 4415^6^), and cycads (*n* = 303^8^). Errors bars show the plausible range for each index value. The stand-alone point for global sharks and rays indicates the 2006 starting point for the global shark and ray RLI^30^. A RLI value of 1 represents a group of entirely Least Concern species while a value of 0 represents an entirely Extinct group. (**b**) Change in IUCN Red List status of Northeast Atlantic sharks and rays between 1980 and 2015 (LC: Least Concern, NT: Near Threatened, VU: Vulnerable, EN: Endangered, CR: Critically Endangered). (**c**) Change in IUCN Red List status of Mediterranean sharks and rays from 1980 to 2015. Colour gradients indicate changing species categorisations between IUCN assessments. Silhouette images were downloaded from phylopic.org and for the bird image specifically we credit Jean-Raphaël Guillaumin and T. Michael Keesey^©^.

We find that sharks and rays in the Northeast Atlantic and Mediterranean Sea faced elevated levels of extinction risk by 1980 and since then their status has steadily worsened (Fig. 1a). The RLI is scaled from zero to one, where zero means all species are Extinct and one means all species are Least Concern^4^. In the Northeast Atlantic the shark and ray RLI declined from a backcast value of 0.80 in 1980 to 0.74 in 2005 and further to 0.72 in 2015 (8% decline in RLI over 35 years; Fig. 1a). To represent the potential range of index values from the group assessed, we generated a confidence interval by bootstrapping (sampling with replacement) the 1980 statuses^31^ (upper and lower confidence interval 0.85–0.75). The change in Northeast Atlantic shark and ray status equates to an average rate of decline of 0.2% year^-1^. The backcast Mediterranean Sea start-point was lower by 1980 than the 2015 Northeast Atlantic end-point, reflecting greater Mediterranean extinction risk. The Mediterranean RLI declined from a backcast value of 0.67 in 1980 (upper and lower confidence interval 0.74–0.61) to 0.59 in 2003, then to 0.54 in 2015 (13% decline in RLI over 35 years; Fig. 1a). In this region, the average rate of decline was 0.4% year^−1^, declining from 0.3% year^−1^ (1980–2003) to 0.4% year^−1^ (2003–2015).

Between 1980 and 2015, the percentage of shark and ray species listed as threatened increased by 12% in the Northeast Atlantic (Fig. 1b) and 18% in the Mediterranean Sea (Fig. 1c). Almost one-third (29%, *n* = 35 of 119) of Northeast Atlantic sharks and rays were backcast into threatened categories in 1980 and this fraction rose to under two-fifths (39%, 46) by 2005^20^ and subsequently to over two-fifths (41%, 49) by 2015^25^ (Fig. 1b). In the Mediterranean Sea, almost half (47%, 34 of 72) of shark and ray species were backcast as threatened in 1980, and this fraction increased to over three-fifths (61%, 44)^19^ by 2003 and further to nearly two-thirds (65%, 47) in 2015^25^. The divergence in extinction risk between ocean basins is apparent in the greatest percentage of Northeast Atlantic species listings being Least Concern (57%, *n* = 68 in 1980 and 45%, *n* = 54 in 2015; Fig. 1b). Conversely, while 32% (*n* = 23, the greatest percentage) of Mediterranean species listings were historically Least Concern in 1980, in 2015, the same percentage (32%, *n* = 23, the greatest percentage) are Critically Endangered. When calculating the RLI, the high weighting given to Critically Endangered species made them a key driver of the lower RLI value in the Mediterranean Sea (Fig. 1c; see Methods for category weights).

The increasing extinction risk of Northeast Atlantic and Mediterranean sharks and rays is greater than any globally assessed vertebrate group on the RLI (Fig. 1a). Further, this risk is increasing faster than for other vertebrate lineages on the RLI: 7–9 times faster than global birds (1% decline in RLI over 26 years), 3–5 times faster than mammals (1% decline in RLI over 13 years), and 2–3 times faster than amphibians (4% decline in RLI over 26 years; Fig. 1a). Northeast Atlantic and Mediterranean sharks and rays are not declining as rapidly as the globally assessed Wedgefishes and Giant Guitarfishes (Family Rhinidae: 46% decline in RLI over 40 years)^26^ or pelagic (oceanic) sharks and rays (30% decline in RLI over 38 years)^32^. To put the high level of threat in context, there are as many threatened sharks and rays in Europe as there are threatened birds, but the threat level is nearly six times greater by percentage (41%, *n* = 56 of 136 sharks and rays threatened versus 7%, *n* = 56 of 792 birds threatened and Extinct^33^). Between 1994 and 2004, European birds were declining at three-quarters of the rate (2% decline in RLI over 10 years) of Northeast Atlantic sharks and rays and half the rate of Mediterranean sharks and rays^34^ (Fig. 1a). Decades of intense conservation effort has successfully reduced global bird declines^13^ and averted extinctions^35^ maintaining them as the least threatened Class assessed on the global RLI^5^. By comparison, quotas in the Northeast Atlantic^17^ and a 1000 m depth limit for trawl and dredge fisheries in the Mediterranean Sea^36^ were implemented only very recently in the early-2000s.

### Disaggregating the Red List Index by primary habitat

**Figure 2:**
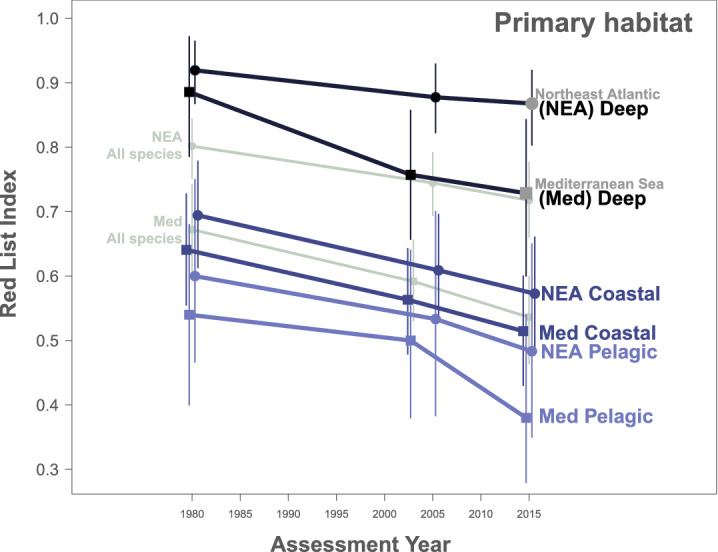
Disaggregating the Red List Index reveals the most at-risk ocean habitats. Red List Indices for deepwater sharks and rays (Northeast Atlantic: *n* = 53 [circles], Mediterranean Sea: *n* = 13 [squares]), coastal and continental shelf species (Northeast Atlantic: *n* = 36, Mediterranean Sea: *n* = 37), and pelagic species (Northeast Atlantic: *n* = 9, Mediterranean Sea: *n* = 10). Light grey lines indicate the regional RLIs for all of these species together (Northeast Atlantic: *n* = 98, Mediterranean Sea: *n* = 60). Confidence intervals represent the range of possible index values. A RLI value of 1 represents a group of entirely Least Concern species while a value of 0 represents an entirely Extinct group.

Overfishing is the major threat identified in the IUCN Red List Assessments facing all Northeast Atlantic and Mediterranean sharks and rays and in essence, these RLIs provide a first comprehensive evaluation of the effect of fishing pressure on marine biodiversity. When the RLIs are partitioned by primary habitat, deepwater species had the lowest-risk RLIs, with the rate of decline slowing by 50% since the early-2000s (Fig. 2, Table 1). This could be a reflection of the expansion of deepwater fishing activity in the late 1980s to early 1990s alongside technological advancement^37^, followed by a reduction in effort in the early-2000s owing to rising fuel costs^38^ and the recent catch prohibitions for deepwater sharks^17^, which was the partial basis of our backcasting decisions. Pelagic species face the highest extinction risk, followed closely by coastal species, although the decline rates are fairly similar for each (Fig. 2, Table 1). Globally, pelagic sharks and rays declined from a backcast RLI value of 0.86 in 1980 to 0.56 in 2018 (*n* = 31)^32^, yet Northeast Atlantic and Mediterranean sharks and rays face a higher risk of extinction than the global average. However, the global rate of decline for pelagic sharks and rays is approximately twice that of this regional analysis, with an average rate of decline of 8% year^−1,^^32^. While the decline in coastal species was consistent throughout the study, pelagic species have declined slightly faster since the early-2000s (Fig. 2, Table 1). These increasing decline rates could be indicative of the need for greater international cooperation between managing bodies for these highly migratory species^39,40^. All three habitat-based groupings reveal higher rates of decline in the Mediterranean Sea than the Northeast Atlantic. We removed the originally Data Deficient species from the disaggregated RLIs, therefore, these trends were not affected by the use of size and depth to predict the statuses of those species (*n* = 21 Northeast Atlantic, *n* = 12 Mediterranean).

**Table 1:** Red List Index values for Northeast Atlantic and Mediterranean Sea sharks and rays between 1980 and 2015.

Region	Scope	*N*	Year	RLI	Decline rate (% year^−1^) 1980–2003/5	Average decline rate (% year^−1^) 1980–2015	Lower bound	Upper bound
					2003/5-2015			
Northeast Atlantic	All species	119	1980	0.80	0.2	0.2	0.75	0.85
			2005	0.74			0.69	0.79
			2015	0.72	0.2		0.66	0.77
	Deep	62	1980	0.92	0.2	0.1	0.86	0.97
			2005	0.88			0.82	0.93
			2015	0.87	0.1		0.81	0.93
	Coast	46	1980	0.70	0.4	0.4	0.61	0.78
			2005	0.61			0.53	0.69
			2015	0.57	0.4		0.49	0.66
	Pelagic	12	1980	0.60	0.3	0.4	0.45	0.77
			2005	0.53			0.37	0.70
			2015	0.48	0.5		0.33	0.65
Mediterranean Sea	All species	72	1980	0.68	0.4	0.4	0.60	0.74
			2003	0.59			0.53	0.66
			2015	0.54	0.4		0.46	0.61
	Deep	14	1980	0.89	0.6	0.5	0.79	0.97
			2003	0.76			0.66	0.86
			2015	0.73	0.3		0.59	0.84
	Coast	49	1980	0.64	0.3	0.3	0.55	0.73
			2003	0.56			0.49	0.65
			2015	0.51	0.3		0.44	0.60
	Pelagic	10	1980	0.54	0.2	0.5	0.42	0.68
			2003	0.50			0.38	0.64
			2015	0.38	1		0.26	0.54

Decline rates are calculated as the annual average between each assessment period, then the overall average taken from both periods.

### Species’ status changes underlying the Red List Index trajectory

**Figure 3:**
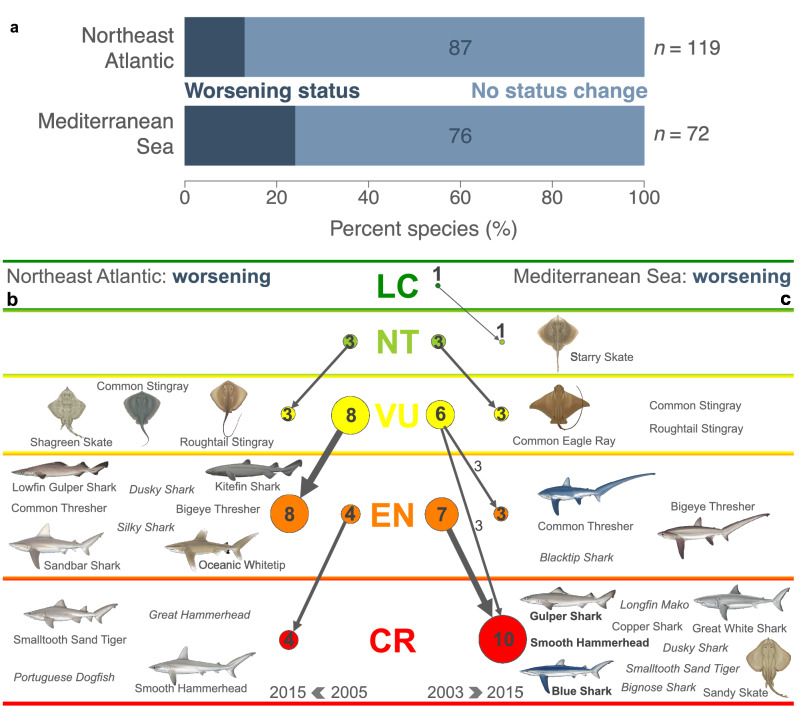
Shark and ray species with worsening status in the Northeast Atlantic Ocean and Mediterranean Sea between 2003/5 and 2015. (**a**) Percentage of sharks and rays in the Northeast Atlantic and Mediterranean Sea with worsening versus static IUCN status between IUCN Red List assessments (Northeast Atlantic 2005–2015, Mediterranean Sea 2003–2015). (**b**) Shark and ray species with worsening status in the Northeast Atlantic between 2005 and 2015 and (**c**) between 2003 and 2015 in the Mediterranean Sea (LC: Least Concern, NT: Near Threatened, VU: Vulnerable, EN: Endangered, CR: Critically Endangered). Bold species names indicate a status change by two IUCN categories between assessments. Italicised species names indicate those for which IUCN status was predicted in previous research^25^. Line thickness (range 1–8) and point size (range 1–10) signifies number of species, as do numbers over each point and next to arrows. Species illustrations by Marc Dando^©^.

Two-thirds of Northeast Atlantic sharks and rays remained in the same IUCN category between 1980 and 2015 (63%, *n* = 75), while over half of all Mediterranean species’ statuses worsened during this time (44%, *n* = 32 remained in the same category in the Mediterranean Sea). In the Northeast Atlantic, 29% (*n* = 35) of species worsened by one IUCN category between the backcast 1980 assessment and 2005 (Fig. 1b) and 13% (*n* = 15) worsened by one category between 2005 and 2015 (Figs. 1b, Fig. 3a + b). Overall, 37% (*n* = 44) of Northeast Atlantic species’ statuses worsened between 1980 and 2015, of which 32% (*n* = 38) worsened by one category and 5% (*n* = 6) worsened by two categories, e.g., Roughtail Stingray *Dasyatis centroura*, Smalltooth Sand Tiger *Odontaspis ferox*, Great Hammerhead *Sphyrna mokorran*, and Smooth Hammerhead *Sphyrna zygaena* (Fig. 1b). In the Mediterranean Sea, 42% (*n* = 30) of species worsened by one category between 1980 and 2003 (Fig. 1c), whereas between 2003 and 2015, 19% (*n* = 14) of species worsened by one category and 4% (*n* = 3) worsened by two categories from Vulnerable to Critically Endangered, e.g., Gulper Shark *Centrophorus granulosus*, Blue Shark *Prionace glauca*, and Smooth Hammerhead (Figs. 1c, and 3a + c). Overall, 56% (*n* = 40) of Mediterranean species’ statuses worsened between 1980 and 2015: 44% (*n* = 32) of species worsened by one category, 8% (*n* = 6) by two categories, e.g., Bignose Shark *Carcharhinus altimus*, Copper Shark *Carcharhinus brachyurus*, Dusky Shark *Carcharhinus obscurus*, and Sandy Skate *Leucoraja circularis* (Fig. 1c). Further, three species worsened by three categories from Near Threatened to Critically Endangered: Gulper Shark, Blue Shark, and Smooth Hammerhead (Fig. 1c). All species with worsening status between 1980 and 2015 have either one or a combination of (1) large body size (> 200 cm total length or disc width), (2) shallow depth distribution (upper depth limit < 500 m), (3) slow life history (generation length > 10 years), or (4) have a range boundary within this region (see Supplementary Fig. S2 and Supplementary spreadsheet). For example, the Mediterranean Sea constitutes the northern limit for Bignose Shark, Copper Shark, Dusky Shark, and Smooth Hammerhead, and Europe is the fringe of the biogeographic distribution of at least seven warmer-water species (e.g. Roughtail Stingray, Smalltooth Sand Tiger, and Great Hammerhead). Species’ sensitivity to both overfishing and climate change is higher at their range boundaries^41,42^, which may explain the greater deterioration of these species’ statuses (Fig. 1b + c).

Although there were six improvements in species status in the Northeast Atlantic and four in the Mediterranean Sea, these changes are not reflected in the RLI slopes because they are ‘non-genuine’^4^. The IUCN defines ‘genuine’ status change as actual change in, e.g., population size between Red List assessments, whereas ‘non-genuine’ changes could be from, e.g., new knowledge or taxonomic changes. ‘Non-genuine’ changes are therefore backcast to retrospectively reflect new knowledge accordingly (see Methods section). For each of the ten improvements, new knowledge revealed the species to be better-off than assessors originally thought in the early-2000s. For example, the incorporation of improved understanding of density-dependent offspring survival into the Northeast Atlantic stock assessment for Spiny Dogfish (*Squalus acanthias*)^43^ resulted in a lower population reduction estimate and a ‘non-genuine’ change in status from Critically Endangered to Endangered. While these improvements were not reflected in the RLI slopes due to retrospective correction of their 2005 status, they did contribute to the overall RLI values. Although these changes did not result from conservation efforts, they are still improvements of scientific knowledge, which supports more accurate species assessment.

### Slow life histories make sharks and rays susceptible to status deterioration

**Figure 4:**
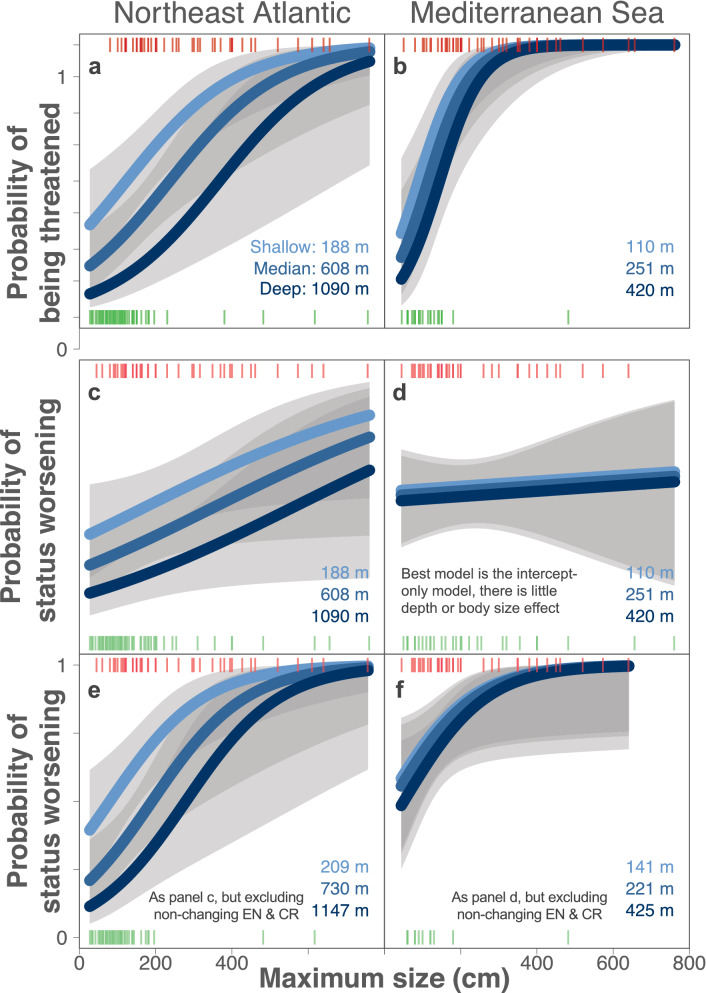
Effect of maximum body size and median depth on Northeast Atlantic Ocean and Mediterranean Sea shark and ray status. The probability that a shark or ray species will be threatened (1: Vulnerable, Endangered, Critically Endangered) or non-threatened (0: Least Concern, Near Threatened) due to the combination of intrinsic biological sensitivity (maximum size, cm total length of sharks, skates, and ghost sharks, or wing span of rays) and exposure to fisheries (median depth, m) in the Northeast Atlantic (a) and Mediterranean Sea (**b**). The probability that a shark or ray’s IUCN status will worsen (1) or stay the same (0) according to the same traits (Northeast Atlantic: **c**, Mediterranean Sea: **d**) and the same test after removing species that remained in the Endangered and Critically Endangered categories from 1980–2015 (Northeast Atlantic: **e**, Mediterranean Sea: **f**). Models are based on 118 Northeast Atlantic and 71 Mediterranean shark and ray species, excluding the outlier Basking Shark (panels **a**–**d**), and 100 and 53 species, respectively (panels **e** + **f**). Lines are the best fits from Generalised Linear Models with IUCN status (**a**,**b**) and status change (**c**–**f**) as the response and maximum size and median depth as the fixed effects (see Supplementary Tables S1, S4, S5). Lines were calculated for the lower (shallow, lightest blue), median, and upper (deep, darkest blue) depth quartiles of each species set. Vertical pink bars represent species with threatened (1; **a**,**b**) or worsening (1; **c**–**f**) status and green bars non-threatened (0; **a**,**b**) or non-changing (0; **c**–**f**) status positioned according to their maximum size.

There is strong consistency in the geographic and biological patterning of shark and ray extinction risk^27–29^. We have previously shown that threatened shark and ray species tend to have slower life histories than non-threatened species (Least Concern and Near Threatened), demonstrated by the relationship between large body size and the likelihood of a shark or ray being listed in a threatened IUCN category^25,30^ (Fig. 4a + b, Supplementary Fig. S1, Supplementary Table S5). Here, we extend this ‘static’ trait-status pattern to show that Northeast Atlantic sharks and rays with *deteriorating* status tend to have slower life histories than species that did not change status between IUCN assessments, although the relationship is weaker for status-change than for static-status (Fig. 4a vs. c, Supplementary Fig. S1, Supplementary Table S1, S2). The relationship between size and changing status is insignificant in the Mediterranean Sea (Fig. 4d, Supplementary Table S1). The intercept-only model fit best, indicating that size and depth do not explain changing status of Mediterranean sharks and rays. This is in part because there are fewer true deepwater shark and ray species in the region (Fig. 4d, Supplementary Table S1).

We have also shown previously that threatened shark and ray species tend to have shallower depth distributions than non-threatened species, which we interpret as the effect of refuge from fishing activity at greater depth^25,30^ (Fig. 4a + b, Supplementary Fig. S1, Supplementary Table S1). Shallower depth also correlates with a likelihood of worsening status in the Northeast Atlantic, for which the top model included both size and depth (Fig. 4c, Supplementary Fig. S1, Supplementary Table S1). These findings were not changed by the inclusion of previously Data Deficient species in our models for which IUCN status was predicted^25^, as the model hierarchy is the same when these species are removed from the analysis (Supplementary Table S1, S3). Further, these patterns are the same when taxonomic family is included as a random effect, though the inclusion of taxonomic family weakened the models in general (Supplementary Tables S1, S3, S4).

The deepening of fishing activity in the early-1990s—particularly in the Northeast Atlantic—has increased the extinction risk of deepwater species over the past 40 years^44^. However, that is not to say that deepwater Northeast Atlantic sharks and rays are more at-risk than shallower species. Rather, shallower species were already at high risk prior to the fishing down of the deepwater species, which reached a similar level of risk during the 1990s. We recognise the recursive nature of this inference, given the prior knowledge of depth offering refuge for sharks and rays^25,30^ and the basis of our backcasting decisions partly being the expansion of fisheries into deeper waters^37^. In the present study, the implicit assumption when backcasting species’ IUCN status is that fishing effort and spatial overlap of fisheries with species’ ranges are highly correlated with the declining abundance of sharks and rays in the Northeast Atlantic and Mediterranean Sea. This is a fundamental assumption of ecological risk assessments. However, the abundance data required to test this assumption are not yet available for sharks and rays in these regions. In future, it would be helpful to test the rigidity of this assumption when these data are available. This would require extensive exploration of the spatial patterning of both fisheries and species, perhaps best undertaken by scientific councils such as the International Council for the Exploration of the Sea’s Working Group for Elasmobranch Fishes.

The consideration of changing-status alone overlooks any species that remained in the Endangered and Critically Endangered categories from 1980–2015 – most notably in the Mediterranean Sea (15%, *n* = 18 of 119 Northeast Atlantic: six remained Endangered and 12 remained Critically Endangered; and 25%, *n* = 18 of 72 Mediterranean: five remained Endangered and 13 remained Critically Endangered, from 1980 to 2015). This substantial percentage of species are all large-bodied and have shallow depth distributions and hence their inertia on the RLI (because they are already nearly as high-risk as can be categorised) washes out the correlation between these traits and changing status, particularly within the Mediterranean data. In fact, once these species are removed from the analysis the probability of sharks and rays having a worsening status based on their maximum size and median depth is very similar to the probability of being threatened (Fig. 4e + f compared with Fig. 4a + b, Supplementary Fig. S1, Supplementary Tables S2, S4, S5). The strength of the models increases considerably when these species are removed and changes the model hierarchy so that both size and depth are related to changing status in both regions, whether taxonomic family is included as a random effect or not (Supplementary Tables S2, S4). This highlights an important consideration: the RLI does not reflect ongoing species population decline unless the decline is sufficient to cross IUCN category thresholds^45^. Hence, these trajectories are likely a conservative representation of true population decline levels in the Northeast Atlantic and Mediterranean Sea.

## Conclusions

### Investing in management to secure a future for overexploited marine species

Sustainable exploitation underpins food security and the well-being of humanity^46^. Many fish stocks have collapsed and while strict management measures have recently reversed some declines in the Northeast Atlantic, insufficient action has been taken to halt or reverse declines in the Mediterranean Sea^24^. There are profound and long-recognised differences between Northeast Atlantic and Mediterranean fisheries management^24,47^. Although numerous solutions to the plight of Mediterranean fisheries have been suggested, few have been effectively implemented for the benefit of sharks and rays^47–49^. Both regions have been heavily fished for centuries^22^, and consequent shifting baselines undermine true levels of decline^50^. The RLIs presented do not convey the considerable degree of population decline and collapse that occurred in the Northeast Atlantic and Mediterranean Sea prior to 1980 as a direct result of overfishing^2,23,51^, nor do the GLMs for changing status (Fig. 4). Drastic improvement of shark- and ray-focused fisheries management is required to deliver on marine biodiversity goals by halting declines and preventing regional species extinctions, particularly in the Mediterranean Sea^24,49,52^. While countries have obligations to improve the status of sharks and rays, improvements in the management of these sensitive species may also yield long-term benefits of more sustainable fisheries for other species, and indeed for human well-being and livelihoods all around the Mediterranean coast too.

Cooperation and higher prioritisation are generally needed to improve the status of sharks^53–55^. Just as the poor status of birds led to the creation of the European ‘Birds Directive’ and extensive improvements in agriculture^56^, the status of sharks and rays reported here would justify the creation of a European ‘Sharks and Rays Directive’ to transform fisheries. Although the European Union’s fisheries management reforms have reversed stock declines of some commercially important teleost species in the Northeast Atlantic^24,52^, we raise five points of concern about fisheries management in the Northeast Atlantic and Mediterranean Sea. First, shark and ray management was only recently implemented in the Northeast Atlantic, while the Mediterranean Sea has essentially been overlooked^24,52,57^. Second, Northeast Atlantic shark and ray protections were implemented only after population collapse, thus recovery could take decades^58^. Third, frequent reports of captured protected species on social media reveal that while catching some species is prohibited, there is often limited awareness among fishers of these protections, especially in the Mediterranean Sea^59^. Fourth, the one measure in place to protect Mediterranean sharks and rays is a ban on fishing below 1000 m depth^36^, but we show here (based on the narrow depth quartiles for Mediterranean species in Fig. 4b + d + f) that this measure is potentially benefiting only a small minority of species at the deepest end of their depth ranges. Finally, there are few resources or funding programs that we are aware of that implement existing protections, with the conservation planning and implementation slack being largely picked-up by Non-Governmental Organisations^16^.

International cooperation between fisheries managers is essential for the benefit of those species that regularly migrate significantly outside the geographic extent considered in the present study (e.g. those listed in Appendix I and II of the Convention on the Conservation of Migratory Species of Wild Animals^60^), as these species are inevitably exploited in waters outside of the jurisdiction of the European Union^53–55^. Indeed, when these migratory species are removed from the RLIs (17%, *n* = 20 from 119 Northeast Atlantic and 22%, *n* = 16 from 72 Mediterranean species), both of the index values improve overall by ~ 5% due to the relatively high extinction risk faced by these migratory and range-boundary species (Supplementary Fig. S2).

### The Northeast Atlantic and Mediterranean Sea could be a ‘canary-in-the-coal mine’

The situation in the Northeast Atlantic and Mediterranean Sea may reflect levels of extinction risk in other regions with equivalent fishing levels and management capacity. For example, aside from seasonal closures, there is relatively little regulation of fisheries in the Arabian Seas and adjacent waters and consequently 51% (*n* = 78 of 153) of sharks and rays are threatened regionally^61^, which is close to the status in the Mediterranean Sea. Without monitoring and periodic evaluation, the local extinction of the more sensitive species may be overlooked, as has already happened frequently, such as for the Angel Shark (*Squatina squatina*), Bramble Shark (*Echinorhinus brucus*), Common Skate complex (*Dipturus batis and D. intermedia*), and other large skates^51, 62–64^. Stock assessments (i.e. species-specific population trend data) of marine fishes—which are largely unavailable for sharks and rays—are typically completed on a smaller scale than IUCN regional or global analyses of extinction risk, although the IUCN Guidelines advise assessors on the use of such data to ensure appropriate proportional representation of stocks within assessment regions^65^. The RLI currently provides the most effective, widely-accepted means of monitoring changing extinction risk, particularly for data-poor groups^66,67^. Our methods are intended for application to the forthcoming global shark and ray RLI, which will eventually reveal how representative the Northeast Atlantic and Mediterranean Sea are of global shark and ray status. Indeed, we know already that the 2014 starting point of the global shark and ray RLI is in a position of greater extinction risk than the latest global Red List assessments for birds, mammals, and corals, but still lower-risk than this regional Red List assessment^30^ (Fig. 1a). Our methods are applicable to any species group assessed by the IUCN more than once, can be extrapolated to any geographic scale, and the implications outlined here are relevant to any species group that is also threatened predominantly by overexploitation.

### Enhancing progress-tracking towards future biodiversity targets

**Figure 5:**
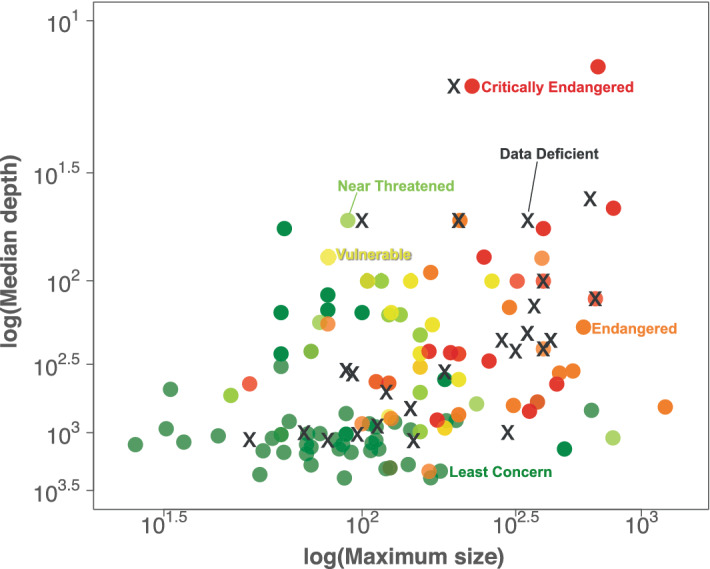
Northeast Atlantic Ocean and Mediterranean Sea Data Deficient sharks and rays have similar trait distribution to data-sufficient species. Relative distribution of IUCN status for *n* = 119 Northeast Atlantic and *n* = 72 Mediterranean shark and ray species based on maximum size (cm, total length for sharks, skates, ghost sharks or wingspan for rays) and median depth (m; as a measure of accessibility to fishing vessels). Circular coloured points represent species assessed as data-sufficient by the IUCN in 2015, while Data Deficient species are marked by X symbols. The Y axis is inverted to represent ocean depth. Point colour corresponds to IUCN status: Critically Endangered—red, Endangered—orange, Vulnerable—yellow, Near Threatened—light green, Least Concern—darker green, and Data Deficient—dark grey.

The exclusion of Data Deficient species from risk indicators undermines global-level reporting on progress towards biodiversity and sustainability targets^68^. Using biological and ecological trait data to incorporate Data Deficient species into extinction risk tracking is currently the most cost-effective, expeditious method for understanding aggregate extinction risk and informing appropriately and efficiently directed species conservation^69^. There was minimal effect on the Northeast Atlantic and Mediterranean RLIs from adding predicted species statuses^25^, reflecting a broad similarity in the biological and ecological trait distributions of the Data Deficient and data-sufficient sharks and rays (Fig. 5). We caution that there are numerous reasons not to expect this similarity in less well-studied regions, where even the most sensitive species may not yet be taxonomically described or assessed but are potentially at risk of extinction^26,70^.

The Convention on Biological Diversity 2020 Aichi targets and the United Nations 2020 Sustainable Development Goals have been missed for Northeast Atlantic and Mediterranean sharks and rays. There may only be a decade or so to realistically halt and reverse global biodiversity loss before the updated Kunming Target deadline^71^. Here, we demonstrate that the Red List Index can incorporate the status predicted for data-poor species or species-groups and the inclusion of these species enables the most complete progress-reporting possible towards imminent deadlines.

## Methods

### Collation of regional IUCN Red List assessments

We consider a total of 127 unique species, including 119 Northeast Atlantic and 72 Mediterranean, with 57 species spanning both regions (hence, 62 exclusively Northeast Atlantic and 15 exclusively Mediterranean). Species spanning both regions are assigned a separate category for each region, many of which are the same. Of the assessed species there are 49 skates and rays (Order Rajiformes), 70 sharks (Order Carcharhiniformes, Hexanchiformes, Lamniformes, Squaliformes, Squatiniformes), and eight ghost sharks (Order Chimaeriformes). This list does not include two species assessed by the IUCN as they are no longer considered breeding residents (Tiger Shark *Galeocerdo cuvier* and Marbled Stingray *Dasyatis marmorata*). Species not breeding in the region are considered ‘vagrant’ according to IUCN terminology^65^. All species included in the analysis were agreed by IUCN Red List assessors to be valid regionally at the 2015 workshop. We classify species according to their primary habitat in accordance with IUCN Red List assessment, allocating those species that span multiple habitats according to the habitat in which they spend the majority of their life cycle. Coastal/continental shelf species are mainly demersal bottom-dwelling (< 200 m depth), with deepwater species predominantly deeper than the continental shelf (≥ 200 m depth), and pelagic species mostly in open, offshore water^30^.

The Northeast Atlantic and Mediterranean Sea were assessed by the IUCN Shark Specialist Group both in 2003/2005^19,20^ and 2015^25^ and the Red List assessments from 2015 were merged to present a regional IUCN Red List assessment to the European Union^21^. All IUCN Red List assessments from 2003, 2005, and 2015 were completed according to the IUCN Categories and Criteria, version 3.1^45^. The 2003/2005 assessments were completed using the first edition of the version 3.1 criteria^72^ and the 2015 assessments using the second edition^45^. The same Red List assessment system is maintained in both editions, the IUCN updated the document in order to adapt and maintain its usefulness as a conservation tool alongside ongoing technological advances in data analysis^45^. The IUCN published an overall status designation for each species when reporting to the European Union^21^ and did not report differences between the Northeast Atlantic and Mediterranean Sea sub-regions. To avoid masking status changes for higher-risk species in the Mediterranean Sea, we went back to the separate sub-region status evaluations completed in the 2014 workshop, rather than the published, combined IUCN Red List (see Supplementary data for the originally reported merged categorisations and all alternative versions of categorisations).

We consider only the five main extant (‘data-sufficient’) IUCN categories in the present study by replacing all Data Deficient listings with the predicted categorisations from previous research^25^. We therefore consider both assessed and predicted listings of Least Concern, Near Threatened, and the three ‘threatened’ categories (in ascending order of threat): Vulnerable, Endangered, and Critically Endangered^65^. The predicted statuses from previous research for all Data Deficient species in the Northeast Atlantic and Mediterranean Sea are considered among IUCN-assessed statuses throughout the present study without differentiation^25^, i.e. the predicted listings have the same influence on the RLI value as the assessed listings and there is no weighting or subjective quality assigned to the predicted statuses. All data-sufficient Northeast Atlantic and Mediterranean sharks and rays were assessed by the IUCN based on population size, under the IUCN’s Criterion A. Criterion A is assigned based on thresholds of percentage population reduction over a three-generation period either ‘estimated’, ‘inferred’, or ‘suspected’ over the greater of ten years or three generation lengths^65^. These IUCN terms (listed in order of decreasing data-certainty) have specific meaning when used in IUCN assessments, acting as sub-categories, and the same meaning is intended in the present study. Whereas estimated declines (and the status categorisation they inform) are typically based on landings and catch data, inferred or suspected declines require some assumption. These categorisations are typically inferred from similar, more data-rich species or either inferred or suspected from information such as geographic and depth range that can be used to discern the degree of overlap with fisheries. A ‘similar’ species would ideally be a congener of similar maximum body size, depth range, generation length, and with the same threats (e.g. Bigeye Thresher Shark *Alopias superciliosus* from Common Thresher Shark *Alopias vulpinus*, see spreadsheet in Supplementary Information). The 2015 Red List reassessments were completed by multiple assessors, peer reviewed by two additional experts, then consistency checked by the Red List Authority to ensure utmost consistency of terminology and that a precautionary view of the evidence was applied across assessments^73^. The variation in subjectivity between assessors in each assessment year is minimised through the application of the IUCN Red List Guidelines^65^, training materials, and guidance on what value system to use when assessing, i.e. precautionary instead of evidentiary. In the early-2000s, the ‘evidentiary approach’ to assessing was more common among fisheries scientists, whereas the ‘precautionary approach’ has been more strictly adopted since the reassessment began in 2014. The evidentiary approach relies almost entirely upon available population data, whereas the precautionary approach encourages the use of all supporting information to determine extinction risk across the spectrum of certainty outlined above, even in the absence of actual population data, and encompassing a wider range of traditional and fishers’ ecological knowledge^16,74–76^. We do not account for the varying degrees of uncertainty statistically and instead treat all listing-certainty, including that of the predicted listings from previous research^25^, as equal in our statistical models. See Supplementary Data for the original IUCN status designations that were retained for analyses.

### Backcast historical categorisations

For IUCN assessments, backcasting is the process of adjusting historical categorisations to reflect new knowledge and was introduced to standardise the process of adding new species to the RLI as well as to retrospectively correct species status^4,7,26^. The basis for backcasting is the same as for writing official IUCN Red List assessments: the pertinent available information is gathered and used to assign an historical extinction risk categorisation using a precautionary approach^4,7,26^. For the present study, this included life history and ecology information from scientific literature, fishing records, reports on trends in fishing effort, and the Red List assessments completed in the early-2000s and 2015^26,32^. The choice of a backcast timeline is a trade-off between effect size (a longer timeline is likely to have a greater difference in status) against the decreasing availability of data (the further back in time we go, less data and knowledge are available). We chose to backcast status to 1980 due to (1) data availability; (2) fishing effort doubling in the Northeast Atlantic and Mediterranean Sea between 1950 and 1980^23^; and (3) deepwater fishing capacity expanding from a maximum depth of ~ 800 m to > 1000 m^37^ in the late-1980s to early-1990s. For most species, the available information allowed a degree of certainty in line with ‘inference’, as per the levels of IUCN-assessment certainty outlined above. For example, the Common Skate complex (*Dipturus batis and D. intermedius*) is known to have been depleted by 1980 due to heavy fishing pressure because a publication in 1981 already warned of its near-extinction^51^. This species was therefore backcast from 2015 and 2005 statuses of Critically Endangered to have already been Critically Endangered in 1980. A similar situation was true for the Angel Shark (*Squatina squatina*)^77,78^. Further, all deepwater species with little or none of their depth range shallower than 800 m depth were inferred to be Least Concern in 1980 unless catch or landings data showed otherwise. This assumption was based on the refuge these species had at depth before deepwater fisheries expanded to depths > 1000 m in the early-1990s^37^, so the degree of certainty is in line with inference. Similarly, all species listed as Least Concern in the early-2000s and 2015 were assumed to have been Least Concern in 1980 as well, as the overall increasing regional fishing effort makes it unlikely that these species would have been higher-risk previously. A more complicated example is the Gulper Shark (*Centrophorus granulosus*) in the Mediterranean Sea, as follows: (1) at least half of this species’ depth range (100–1490 m) has overlapped with fishing activity since the 1950s; (2) it has a very long 20-year generation length and consequent slow population turnover; (3) it was listed as Vulnerable in 2003 after deepwater fishing effort increased considerably in the 1990s and was therefore likely lower-risk prior to the 1990s; but (4) it is unlikely that this species was Least Concern in 1980 due to the combination of points (1) and (2). Hence, we backcast the 1980 status for Gulper Shark as Near Threatened. For all other species-specific justifications for backcasting of status and the supporting data and literature, please refer to the spreadsheet in the Supplementary Information. We note that the process of backcasting has few choices. For example, a species listed as Vulnerable in 2015 and Near Threatened in 2005 must have been either Near Threatened or Least Concern in 1980 based on our knowledge of fishing trends. To conservatively account for the uncertainty around the 1980 categorisations we used the ‘red’ package in R to calculate confidence intervals for the RLI values by bootstrapping 1000 iterations of each status assignment^31^. The confidence intervals generated do not account for the uncertainty in assigned status of individual species, rather they show the range of possible values for the RLI of the species group as determined from the group as it was assessed. Therefore, the bootstrapping assumes that the status designations are representative of sharks and rays in the region.

The early-2000s Red List assessments were originally highly conservative because of the evidentiary mindset that was prevalent among fisheries scientists at the time. Since the early-2000s, knowledge of fisheries target and limit reference points has improved owing to empirical and simulations analyses of numerous teleost and elasmobranch populations^24,79^, as well as the precautionary approach being consistently adopted during the later Red List assessments. We therefore backcast several of the 2003/2005 statuses to be in-line with this new knowledge and the precautionary approach (see spreadsheet in Supplementary Information). For example, the Common Thresher Shark had originally been listed as Near Threatened in the Northeast Atlantic in 2005 using an evidentiary approach. The 2015 status was decided as Endangered using the precautionary approach, which created an unrealistically steep decline from Near Threatened to Endangered over the ten years between 2005 and 2015. In this case, the 2005 status was backcast as Vulnerable based on new knowledge of the species’ general decline, particularly the fact that fishing effort across this species’ range doubled between 1950 and 1980 and was comparably stable between 2005 and 2015^23^. The majority of population decline was therefore likely to have occurred long before 2005 (see spreadsheet in Supplementary Information for justification of statuses altered from the early-2000s).

We also conservatively backcast species that were listed as Data Deficient in 2003/2005 but became data-sufficient by 2015, as per the standard backcasting protocol adopted by the IUCN^4^. These species were backcast under the same category as the 2015 IUCN Red List assessment unless evidence was available of ‘genuine’ improvement or deterioration in status of sufficient magnitude to cross IUCN category thresholds since the early-2000s^4^. Whereas a genuine species status improvement (not seen in this study) might result from successful conservation efforts, ‘non-genuine’ improvements that occurred between the early-2000s and 2015 were a result of, for example, gained knowledge that revealed the previous assessment to be overly evidentiary and insufficiently precautionary^80^. Most category changes between IUCN assessments are non-genuine due to new information becoming available, hence, due to backcasting they are not reflected in the RLI slope. The same backcasting approach as outlined above was used in these instances (see spreadsheet in Supplementary Information for comparison of all original and backcast statuses).

### Calculating the extinction risk trajectory: the Red List Index

We calculated RLIs from the 119 Northeast Atlantic and 72 Mediterranean shark and ray Red List assessments from 2003/2005 and 2015, and the 1980 backcast. A RLI value is calculated for each assessment year based on the proportion of species listed in each IUCN Red List category. The point of the RLI is to show the overall change in extinction risk over time (i.e. extinction risk trajectory) and it cannot be reliably interpreted otherwise, i.e. it does not convey the proportion of published listings in each category at any given time. For the calculation of each RLI value, the IUCN Red List categories are weighted such that the greater the threat category, the greater the weight: Least Concern = 0, Near Threatened = 1, Vulnerable = 2, Endangered = 3, Critically Endangered = 4, and (Regionally) Extinct (EX) = 5^4^ (there were no Regionally Extinct species to consider in the present study). Each category change between assessment years is equally weighted such that a status change from Least Concern to Near Threatened is equal to changing from Critically Endangered to Extinct in terms of its overall influence on the RLI slope^4^. The decline between categories is therefore linear and the decline rate of the index is dependent on the number of categories considered. The RLI value of a particular year (*t*) is calculated by multiplying the sum of all species (*s*) in each IUCN category by the relevant category weight (*W*), then summing the product and dividing by the maximum possible product (number of species: *N*, multiplied by the maximum category weight: *W*_*EX*_ = 5)^4^. Finally, this value is subtracted from one to give an index value between zero (all species are Extinct) and one (all species are Least Concern)^4^. This calculation is completed separately for each assessment year under consideration. The RLI is represented by Eq. (1)^4^:

1 $$RLI_{t} = 1 - \frac{{\mathop \sum \nolimits_{s} W_{{c\left( {t,s} \right)}} }}{{W_{EX} \cdot N}}$$

### Testing for a differential effect of size and depth on worsening IUCN Red List status

We then tested whether changes in IUCN status were related to biological or ecological traits of assessed species, reflecting the prior knowledge that intrinsic sensitivity to fishing pressure, combined with the degree of exposure to fishing activity, is functionally related to IUCN status^25,30^. We ran binomial Generalized Linear Models (GLMs) using the glm function in R version 3.5.2^81^ with maximum size and median depth as fixed effects and IUCN status (non-threatened = 0, threatened = 1) or status change (no status change/improving status = 0, worsening status = 1) as the response. We also ran equivalent Generalized Linear Mixed-effects Models (GLMMs) with binomial error and a logit link using the glmer function in the lme4 package^82^, including taxonomic family as a random effect to account for phylogenetic covariation^83,84^. We also ran the models for each region (a) replacing the response variable with ‘2015 static IUCN status’ (*n* = 118 Northeast Atlantic and *n* = 71 Mediterranean species in both sets of models, excluding the outlier Basking Shark *Cetorhinus maximus*), and (b) excluding all species that remained in either the Endangered or Critically Endangered categories from 1980 to 2015 (*n* = 100 Northeast Atlantic and *n* = 53 Mediterranean species), for comparison. We modelled two biological and ecological traits (fixed effects) to discern differing threat levels between the Northeast Atlantic and Mediterranean Sea: maximum body size as a measure of intrinsic sensitivity and median depth as a measure of exposure to fishing activity. The working model is represented by Eq. (2):

2 $${\text{log}}\left( {\frac{{p_{i} }}{1} - p_{i} } \right)^{ } = \beta_{0} + \beta_{i,j} X_{i,j} + \beta_{i,k} X_{i,k}$$

where the probability of a species (*i*) having ‘worsening IUCN status’ is assumed to be binomially distributed with a mean *p*_*i*_, where *β*_0_ is the coefficient estimate for the intercept, *β*_i,j_ and *β*_i,k_ are the fitted coefficients for maximum size (*j*) and median depth (*k*), and *X*_i,j_ and *X*_i,k_ are the trait values of *j* and *k* for species *i*^83,84^. We compiled all data from the regional IUCN Red List assessments completed in 2015^18,21^. We centred and scaled size and depth by two standard deviations^85^ so that the effect size of these continuous variables was equivalent to a binary predictor, allowing us to directly compare them to the binary status change. The probabilities used to plot the lines in Fig. 4 were extracted manually in R by exponentiating the log(probability) from Eq. (2) for each species. We used the Akaike Information Criterion corrected for smaller sample sizes (AIC_c_)^86^ and ranked models according to their delta AIC^87^ (zero is the highest ranked model with any model two or fewer AIC units away from zero not significantly different from the best). To evaluate model fit, we used the MuMIn package version 1.43.15^88^ in R to calculate marginal and conditional R-squared values for the GLMMs and pseudo-R-squared values for the GLMs. We tested for collinearity between maximum body size and median depth by calculating the variance inflation factor (vif) using the car package version 3.0-7^89^ in R. The values for both regions fell below three (both were close to one), which is typically used as an upper limit^90^, indicating acceptably low collinearity for inclusion of both variables in the models.

## Supplementary Information

Supplementary Information 1.

Supplementary Information 2.

## Data Availability

The original data used in this manuscript are publicly available at iucnredlist.org. We have also provided a summary spreadsheet of all of the data in the Supplementary Information.

## References

[1] McClenachan L, Cooper AB, Carpenter KE, Dulvy NK (2011). Extinction risk and bottlenecks in the conservation of charismatic marine species. Conserv. Lett..

[2] Dulvy NK, Jennings S, Rogers SI, Maxwell DL (2006). Threat and decline in fishes: An indicator of marine biodiversity. Can. J. Fish. Aquat. Sci..

[3] CBD & UNEP. *Strategic Plan for Biodiversity 2011–2020 and the Aichi Targets ‘Living in Harmony with Nature’*. 2pp. https://www.cbd.int/doc/strategic-plan/2011-2020/Aichi-Targets-EN.pdf (Secretariat of the Convention on Biological Diversity, Montreal, Quebec, 2011).

[4] Butchart SHM (2007). Improvements to the Red List Index. PLoS ONE.

[5] Butchart SHM (2004). Measuring global trends in the status of biodiversity: Red List Indices for birds. PLoS Biol..

[6] Butchart SHM (2005). Using Red List Indices to measure progress towards the 2010 target and beyond. Philos. Trans. R. Soc. B Biol. Sci..

[7] Hoffmann M (2011). The changing fates of the world’s mammals. Philos. Trans. R. Soc. B Biol. Sci..

[8] Marler PN, Marler TE (2015). An assessment of Red List data for the cycadales. Trop. Conserv. Sci..

[9] Carpenter KE (2008). One-third of reef-building corals face elevated extinction risk from climate change and local impacts. Science (80-. ).

[10] Gärdenfors U (2001). Classifying threatened species at national versus global levels. Trends Ecol. Evol..

[11] Szabo JK, Butchart SHM, Possingham HP, Garnett ST (2012). Adapting global biodiversity indicators to the national scale: A Red List Index for Australian birds. Biol. Conserv..

[12] Juslén A, Hyvärinen E, Virtanen LK (2013). Application of the Red-List Index at a national level for multiple species groups. Conserv. Biol..

[13] Hoffmann M (2010). The impact of conservation on the status of the world’s vertebrates. Science (80-. ).

[14] IPBES. *Summary for Policymakers of the Global Assessment Report on Biodiversity and Ecosystem Services of the Intergovernmental Science-Policy Platform on Biodiversity and Ecosystem Services*. 56pp. https://ipbes.net/document-library-catalogue/summary-policymakers-global-assessment-laid-out (IPBES Secretariat, Bonn, Germany, 2019).

[15] Dulvy NK, Sadovy Y, Reynolds JD (2003). Extinction vulnerability in marine populations. Fish Fish..

[16] Lawson JM (2020). Global extinction risk and conservation of Critically Endangered angel sharks in the Eastern Atlantic and Mediterranean Sea. Int. Counc. Explor. Seas J. Mar. Sci..

[17] WGEF. *Report of the Working Group on Elasmobranch Fishes (WGEF)*. 671pp. https://www.ices.dk/community/groups/pages/wgef.aspx (ICES CM, Lisbon, Portugal, 2018).

[18] Dulvy, N. K., Allen, D. J., Ralph, G. M. & Walls, R. H. L. *The conservation status of sharks, rays and chimaeras in the Mediterranean Sea*. 14pp. https://portals.iucn.org/library/node/47636 (IUCN, Malaga, Spain, 2016)

[19] Cavanagh RD, Gibson C (2007). Overview of the Conservation Status of Cartilaginous Fishes (Chondrichthyans) in the Mediterranean Sea.

[20] Gibson C, Valenti SV, Fowler SL, Fordham SV (2008). The Conservation Status of Northeast Atlantic Chondrichthyans: Report of the IUCN Shark Specialist Group Northeast Atlantic Regional Red List Workshop.

[21] Nieto, A. *et al. European Red List of Marine Fishes.* 88pp. 10.2779/082723 (Publications Office of the European Union, Luxembourg, 2015).

[22] Barrett JH, Locker AM, Roberts CM (2004). The origins of intensive marine fishing in medieval Europe: The English evidence. Proc. R. Soc. B Biol. Sci..

[23] Rousseau Y, Watson RA, Blanchard JL, Fulton EA (2019). Evolution of global marine fishing fleets and the response of fished resources. Proc. Natl. Acad. Sci. U.S.A..

[24] Fernandes PG (2017). Coherent assessments of Europe’s marine fishes show regional divergence and megafauna loss. Nat. Ecol. Evol..

[25] Walls RHL, Dulvy NK (2020). Eliminating the dark matter of data deficiency by predicting the conservation status of Northeast Atlantic and Mediterranean Sea sharks and rays. Biol. Conserv..

[26] Kyne PM (2020). The thin edge of the wedge: Extremely high extinction risk in wedgefishes and giant guitarfishes. Aquat. Conserv. Mar. Freshw. Ecosyst..

[27] Jennings S, Reynolds JD, Mills SC (1998). Life history correlates of responses to fisheries exploitation. Proc. R. Soc. B Biol. Sci..

[28] Frisk MG, Miller TJ, Fogarty MJ (2001). Estimation and analysis of biological parameters in elasmobranch fishes: A comparative life history study. Can. J. Fish. Aquat. Sci..

[29] Hutchings JA, Myers RA, García VB, Lucifora LO, Kuparinen A (2012). Life-history correlates of extinction risk and recovery potential. Ecol. Appl..

[30] Dulvy NK (2014). Extinction risk and conservation of the world’s sharks and rays. Elife.

[31] Cardoso, P. Package ‘red’. 32pp. Available at: https://cran.r-project.org/web/packages/red/red.pdf (2020).

[32] Pacoureau N (2021). Half a century of global decline in oceanic sharks and rays. Nature.

[33] IUCN 2021. *The IUCN Red List of Threatened Species. Version 2021–1*. Web page: https://www.iucnredlist.org. Accessed 7 April 2021.

[34] European Environment Agency. *Red List Index for European species.* 23pp. https://www.eea.europa.eu/data-and-maps/indicators/red-list-index-for-european-species/red-list-index-for-european (EEA, Copenhagen, Denmark, 2010).

[35] Bolam FC (2020). How many bird and mammal extinctions has recent conservation action prevented?. bioRxiv.

[36] GFCM. *Recommendation GFCM/29/2005/1 on the Management of Certain Fisheries Exploiting Demersal and Deep-Water Species and the Establishment of a Fisheries Restricted Area Below 1000 m*. 2pp. https://www.cbd.int/doc/meetings/mar/soiom-2016-01/other/soiom-2016-01-gfcm-02-en.pdf (2005).

[37] Morato T, Watson R, Pitcher TJ, Pauly D (2006). Fishing down the deep. Fish Fish..

[38] Abernethy KE, Trebilcock P, Kebede B, Allison EH, Dulvy NK (2010). Fuelling the decline in UK fishing communities?. ICES J. Mar. Sci..

[39] Campana SE (2016). Transboundary movements, unmonitored fishing mortality, and ineffective international fisheries management pose risks for pelagic sharks in the Northwest Atlantic. Can. J. Fish. Aquat. Sci..

[40] Queiroz N (2019). Global spatial risk assessment of sharks under the footprint of fisheries. Nature.

[41] Fredston-Hermann A, Selden R, Pinsky M, Gaines SD, Halpern BS (2020). Cold range edges of marine fishes track climate change better than warm edges. Glob. Change Biol..

[42] Yan HF (2021). Overfishing and habitat loss drives range contraction of iconic marine fishes to near extinction. Sci. Adv..

[43] De Oliveira JA, Ellis JR, Dobby H (2013). Incorporating density dependence in pup production in a stock assessment of NE Atlantic spurdog *Squalus acanthias*. ICES J. Mar. Sci..

[44] Bailey DM, Collins MA, Gordon JDM, Zuur AF, Priede IG (2009). Long-term changes in deep-water fish populations in the northeast Atlantic: A deeper reaching effect of fisheries?. Proc. R. Soc. B Biol. Sci..

[45] IUCN. *IUCN Red List Categories and Criteria: Version 3.1*. Second Edition. iv + 32pp. https://www.iucnredlist.org/resources/grid (IUCN, Gland, Switzerland and Cambridge, UK, 2012).

[46] Godfray HCJ (2010). Food security: The challenge of feeding 9 billion people. Science (80-. ).

[47] Smith ADM, Garcia SM (2014). Fishery management: Contrasts in the Mediterranean and the Atlantic. Curr. Biol..

[48] Caddy JF (2009). Practical issues in choosing a framework for resource assessment and management of Mediterranean and Black Sea fisheries. Mediterr. Mar. Sci..

[49] Colloca F (2013). Rebuilding Mediterranean fisheries: A new paradigm for ecological sustainability. Fish Fish..

[50] Pauly D (1995). Anecdotes and the shifting baseline syndrome of fisheries. Trends Ecol. Evol..

[51] Brander K (1981). Disappearance of common skate Raia batis from Irish Sea. Nature.

[52] Vasilakopoulos P, Maravelias CD, Tserpes G (2014). The alarming decline of Mediterranean fish stocks. Curr. Biol..

[53] Oliver S, Braccini M, Newman SJ, Harvey ES (2015). Global patterns in the bycatch of sharks and rays. Mar. Policy.

[54] Harrison AL (2018). The political biogeography of migratory marine predators. Nat. Ecol. Evol..

[55] White C, Costello C (2014). Close the high seas to fishing?. PLoS Biol..

[56] Donald PF (2007). International conservation policy delivers benefits for birds in Europe. Science (80-. ).

[57] Demirel N, Zengin M, Ulman A (2020). First large-scale eastern Mediterranean and black sea stock assessment reveals a dramatic decline. Front. Mar. Sci..

[58] Clarke MW (2009). Sharks, skates and rays in the northeast Atlantic: Population status, advice and management. J. Appl. Ichthyol..

[59] Abudaya M (2018). Speak of the devil ray (*Mobula mobular*) fishery in Gaza. Rev. Fish Biol. Fish..

[60] CMS. *Appendices I and II of the Convention on the Conservation of Migratory Species of Wild Animals (CMS)*. 16pp. Available at: https://www.cms.int/en/species/appendix-i-ii-cms (2020).

[61] Jabado RW (2017). The Conservation Status of Sharks, Rays, and Chimaeras in the Arabian Sea and Adjacent Waters.

[62] Iglésias SP, Toulhoat L, Sellos DY (2010). Taxonomic confusion and market mislabelling of threatened skates: Important consequences for their conservation status. Aquat. Conserv. Mar. Freshw. Ecosyst..

[63] Ferretti F, Myers RA, Serena F, Lotze HK (2008). Loss of large predatory sharks from the Mediterranean Sea. Conserv. Biol..

[64] Iglésias SP, Mollen FH (2018). Cold case: The early disappearance of the Bramble shark (*Echinorhinus brucus*) in European and adjacent waters. Oceans Past News.

[65] IUCN (2012). Guidelines for Application of IUCN Red List Criteria at Regional and National Levels: Version 4.0.

[66] CBD.* Indicators for Assessing Progress Towards the 2010 Target: Change in Status of Threatened Species*. Convention on Biological Diversity, UNEP/CBD/AHTEG-2010-Ind/1/INF/9. 10pp. Available at: https://www.cbd.int/meetings/TEGIND-01 (2004).

[67] Brooks TM (2015). Harnessing biodiversity and conservation knowledge products to track the Aichi targets and sustainable development goals. Biodiversity.

[68] Bland LM (2017). Toward reassessing data-deficient species. Conserv. Biol..

[69] Bland LM (2015). Cost-effective assessment of extinction risk with limited information. J. Appl. Ecol..

[70] White WT, Kyne PM, Harris M (2019). Lost before found: A new species of whaler shark *Carcharhinus obsolerus* from the Western Central Pacific known only from historic records. PLoS ONE.

[71] Mace GM (2018). Aiming higher to bend the curve of biodiversity loss. Nat. Sustain..

[72] IUCN (2001). IUCN Red List Categories and Criteria: Version 3.1. IUCN Species Survival Commission.

[73] Regan TJ (2005). The consistency of extinction risk classification protocols. Conserv. Biol..

[74] Hiddink JG, Shepperson J, Bater R, Goonesekera D, Dulvy NK (2019). Near disappearance of the Angelshark *Squatina squatina* over half a century of observations. Conserv. Sci. Pract..

[75] Bom RA, van de Water M, Camphuysen KCJ, van der Veer HW, van Leeuwen A (2020). The historical ecology and demise of the iconic Angelshark *Squatina squatina* in the southern North Sea. Mar. Biol..

[76] Shephard S, Wögerbauer C, Green P, Ellis JR, Roche WK (2019). Angling records track the near extirpation of angel shark *Squatina squatina* from two Irish hotspots. Endanger. Species Res..

[77] Martin CS (2010). Spatio-temporal patterns in demersal elasmobranchs from trawl surveys in the eastern English Channel (1988–2008). Mar. Ecol. Prog. Ser..

[78] Burt, G. J., Ellis, J. R., Harley, B. F. & Kupschus, S. *The FV Carhelmar Beam Trawl Survey of the Western English Channel (1989–2011): History of the Survey, Data Availability and the Distribution and Relative Abundance of Fish and Commercial Shellfish. CEFAS, Norwich, UK. Science Series, Technical Report no. 151. 139pp.* (2013).

[79] Marandel F, Lorance P, Trenkel VM (2019). Determining long-term changes in a skate assemblage with aggregated landings and limited species data. Fish. Manag. Ecol..

[80] IUCN Standards and Petitions Subcommittee. *Guidelines for Using the IUCN Red List Categories and Criteria, Version 11. Prepared by the Standards and Petitions Subcommittee.* 87pp. Available at: http://www.iucnredlist.org/documents/RedListGuidelines.pdf (2014).

[81] R Core Team. *R: A Language and Environment for Statistical Computing*. R Foundation for Statistical Computing, Vienna, Austria. Available at: https://www.r-project.org/. (2018).

[82] Bates, D. *et al.* Package ‘lme4’. 126pp. Available at: https://cran.r-project.org/web/packages/lme4/index.html (2020).

[83] Zuur AF, Ieno EN, Walker NJ, Saveliev A, Smith GM (2009). Mixed Effects Models and Extensions in Ecology with R Statistics for Biology and Health.

[84] Zuur AF, Hilbe JM, Ieno EN (2013). A Beginner’s Guide to GLM and GLMM with R: A Frequentist and Bayesian Perspective for Ecologists.

[85] Gelman A (2008). Scaling regression inputs by dividing by two standard deviations. Stat. Med..

[86] Hurvich CM, Tsai C-L (1989). Regression and time series model selection in small samples. Biometrika.

[87] Johnson JB, Omland KS (2004). Model selection in ecology and evolution. Trends Ecol. Evol..

[88] Bartoń, K. Package ‘MuMIn’. 75pp. Available at: https://cran.r-project.org/web/packages/MuMIn/index.html (2019).

[89] John, A. *et al.* Package ‘car’. 149pp. Available at: https://cran.r-project.org/web/packages/car/index.html (2020).

[90] Zuur AF, Ieno EN, Smith GM (2007). Analysing Ecological Data. Statistics for Biology and Health.

