# Supplementary material for: Tracking the rising extinction risk of sharks and rays in the Northeast Atlantic Ocean and Mediterranean Sea
Source: Sci Rep. 2021 Jul 28;11:15397. doi: 10.1038/s41598-021-94632-4 (PMC8319307; doi:10.1038/s41598-021-94632-4)
Supplement: Supplementary file 1 — Supplementary Information 1. [file 41598_2021_94632_MOESM1_ESM.docx]

**­Supplementary Material**

Title: Tracking the rising extinction risk of sharks and rays in the Northeast Atlantic Ocean and Mediterranean Sea

Authors: Rachel HL Walls^1^* and Nicholas K Dulvy^1^

^1^Earth to Ocean Research Group, Department of Biological Sciences, Simon Fraser University, Burnaby, BC, V5A 1S6, Canada

*Correspondence: Rachel HL Walls, +1(778)251-8227, email: rwalls@sfu.ca

This MS Word file includes:

Supplementary Table S1 | Model selection 4

Supplementary Table S2 | R-squared values for plotted models in Figure 4 5

Supplementary Table S3 | Model selection excluding originally Data Deficient species 6

Supplementary Table S4 | Model selection excluding static-status Endangered and Critically Endangered species

from 1980–2015 7

Supplementary Table S5 | Model outputs for 2015 static IUCN status 8

Supplementary Figure S1 | Effect of body size and depth distribution on IUCN status in Northeast Atlantic and

Mediterranean sharks and rays 9

Supplementary Figure S2 | Various effects on the Red List Indices from removing potentially influential species groups 10

Supplementary Figure S3 | Model diagnostics for Northeast Atlantic Generalised Linear Model plotted in Figure 4c 11

Supplementary Figure S4 | Model diagnostics of standard residuals for Northeast Atlantic Generalised Linear Model plotted

in Figure 4c 12

Supplementary Figure S5 | Model diagnostics of outliers for Northeast Atlantic Generalised Linear Model plotted in

Figure 4c 13

Supplementary References 13

The separate MS Excel spreadsheet includes:

**All data |** Including original IUCN species categorisations from merged European Union assessment, all other original/backcast/predicted species categorisations for each region and year, species-specific justifications for backcasting, life history and ecological data, reference list.

**Supplementary Table S1 | Model selection.** Generalised Linear Models and Generalised Linear Mixed-Effects Models testing the effect of maximum body size (cm, total length/wing span) and median depth (m) on whether a shark or ray species’ IUCN status is worsening (1) or not (0) in the Northeast Atlantic (*n* = 118) and Mediterranean Sea (*n* = 71). Models exclude the outlier Basking Shark. (*K* = degrees of freedom, logLik = negative log likelihood, AIC_c_ = AIC corrected for small sample size, ΔAIC = model ranking [0 is best, anything <2 is not significantly different from best, bold], AIC wt = AIC weight, Coef est = coefficient estimates, Ran effect var = variance of random effect [only applicable to c] , SD = standard deviation). Grey text represents models that have the same negative log likelihood as less complex models, hence they are removed from the calculation of AIC weights.

| **Model** | **Northeast Atlantic** | | | | | | | **Mediterranean Sea** | | | | | | |
| --- | --- | --- | --- | --- | --- | --- | --- | --- | --- | --- | --- | --- | --- | --- |
| **Response:**  Status Change  **Fixed effects** | ***K*** | **logLik** | **AIC_c_** | **ΔAIC** | **AIC wt** | **Coef**  **est** | **Ran effect var** | ***K*** | **logLik** | **AIC_c_** | **ΔAIC** | **AIC wt** | **Coef**  **est** | **Ran effect var** |
| 1. **Without accounting for taxonomic family (GLM)** | | | | | | | | | | | | | | |
| 1. Intercept | 117 | -78.4 | 158.92 | 19.66 | 4.57e-5 | -0.48 | - | 70 | -48.9 | 99.82 | 0.00 | 0.23 | 0.20 | - |
| 2. Maximum Size | 116 | -69.9 | 143.94 | 4.55 | 8.70e-2 | -0.55 + 1.70 | - | 69 | -48.5 | 101.36 | 1.34 | 0.12 | 0.20 + 0.39 | - |
| 3. Median Depth | 116 | -70.9 | 145.89 | 6.51 | 3.27e-2 | -0.59 + -1.74 | - | 69 | -48.6 | 101.45 | 1.42 | 0.11 | 0.20 + -0.37 | - |
| 4. Max Size + Med Depth | 115 | -66.6 | 139.59 | 0.00 | 8.47e-1 | -0.61 +  1.31 +  -1.24 | - | 68 | -48.3 | 103.23 | 2.87 | 0.06 | 0.20 + 0.36 +  -0.33 | - |
| 1. **Accounting for taxonomic family as a fixed effect (GLM)** | | | | | | | | | | | | | | |
| 5. Family | 88 | -48.9 | 199.92 | 18.63 | 7.61e-5 | - | - | 46 | -27.8 | 162.22 | 5.82 | 0.01 | - | - |
| 6. Maximum Size + Family | 87 | -45.6 | 198.64 | 14.00 | 7.71e-4 | - | - | 45 | -27.6 | 169.78 | 7.41 | 0.01 | - | - |
| 7. Median Depth + Family | 87 | -46.3 | 199.99 | 15.35 | 3.93e-4 | - | - | 45 | -25.2 | 165.03 | 2.65 | 0.06 | - | - |
| 8. Max Size + Med Depth + Family | 86 | -43.3 | 199.61 | 11.45 | 2.76e-3 | - | - | 44 | -25.1 | 173.28 | 4.47 | 0.02 | - | - |
| 1. **Accounting for taxonomic family as a random effect (GLMM)** | | | | | | | | | | | | | | |
| 9. (1\|Family) | 116 | -76.1 | 156.35 | 17.08 | 1.66e-4 | -0.36 | 0.96 / 0.98 | 69 | -48.1 | 100.19 | 0.39 | 0.19 | 0.26 | 0.64 / 0.80 |
| 10. Maximum Size + (1\|Family) | 115 | -69.9 | 145.97 | 6.55 | NA | -0.55 + 1.70 | 4e-14 / 2e-7 | 68 | -48.0 | 102.29 | 2.26 | 0.08 | 0.25 + 0.23 | 0.57 / 0.75 |
| 11. Median Depth + (1\|Family) | 115 | -70.0 | 146.07 | 6.73 | 2.93e-2 | -0.52 + -1.84 | 0.41 / 0.64 | 68 | -47.6 | 101.59 | 1.53 | 0.11 | 0.29 + -0.55 | 0.78 / 0.88 |
| 12. Max Size + Med Depth + (1\|Family) | 114 | -66.6 | 141.57 | 1.97 | NA | -0.60 + 1.29 +  -1.28 | 0.07 / 0.26 | 67 | -47.6 | 103.83 | 3.48 | NA | 0.28 + 0.14 +  -0.52 | 0.72 / 0.85 |

**Supplementary Table S2 | R-squared values for plotted models in Figure 4.** Generalised Linear Models testing the effect of maximum body size (cm, total length/wing span) and median depth (m) on whether a shark or ray species’ IUCN status is threatened/worsening (1) or not (0) in the Northeast Atlantic (*n* = 118, or 100 without Endangered [EN] and Critically Endangered [CR] species) and Mediterranean Sea (*n* = 71, or 53 without EN and CR species). Models exclude the outlier Basking Shark. This was the top model for the Northeast Atlantic but the top model for the Mediterranean Sea was the intercept-only model and the models listed here are therefore for the purpose of comparison only.

| **Region** | **Model** | **Pseudo-Rsq**  **(GLM)** | **Marginal Rsq (GLMM)** | **Conditional Rsq (GLMM)** |
| --- | --- | --- | --- | --- |
| **Northeast Atlantic** | 2015 status ~ maximum size + median depth | **0.55** | **-** | **-** |
|  | Status change ~ maximum size + median depth | **0.25** | **-** | **-** |
|  | Status change ~ maximum size + median depth (without static-status EN and CR species) | **0.45** | **-** | **-** |
| **Mediterranean Sea** | 2015 status ~ maximum size + median depth | **0.59** | **-** | **-** |
|  | Status change ~ maximum size + median depth | **0.02** | **-** | **-** |
|  | Status change ~ maximum size + median depth (without static-status EN and CR species) | **0.26** | **-** | **-** |

**Supplementary Table S3 | Model selection excluding originally Data Deficient species.** Generalised Linear Models and Generalised Linear Mixed-Effects Models testing the effect of maximum body size (cm, total length/wing span) and median depth (m) on whether a shark or ray species’ IUCN status is worsening (1) or not (0) in the Northeast Atlantic (*n* = 97) and Mediterranean Sea (*n* = 59). All Data Deficient species (*n* = 21 Northeast Atlantic and *n* = 12 Mediterranean) and the outlier Basking Shark are excluded. (*K* = degrees of freedom, logLik = negative log likelihood, AIC_c_ = AIC corrected for small sample size, ΔAIC = model ranking [0 is best, anything <2 is not significantly different from best, bold], AIC wt = AIC weight, Coef est = coefficient estimates, Ran effect var = variance of random effect [only applicable to c], SD = standard deviation). Grey text represents models that have the same negative log likelihood as less complex models, hence they are removed from the calculation of AIC weights.

| **Model** | **Northeast Atlantic** | | | | | | | **Mediterranean Sea** | | | | | | |
| --- | --- | --- | --- | --- | --- | --- | --- | --- | --- | --- | --- | --- | --- | --- |
| **Response:** Status Change  **Fixed effects:** | ***K*** | **logLik** | **AIC_c_** | **ΔAIC** | **AIC wt** | **Coef**  **est** | **Ran effect var / SD** | ***K*** | **logLik** | **AIC_c_** | **ΔAIC** | **AIC wt** | **Coef est** | **Ran effect var / SD** |
| 1. **Without accounting for taxonomic family (GLM)** | | | | | | | | | | | | | | |
| 1. Intercept | 96 | -60.8 | 123.61 | 7.98 | 9.90e-3 | -0.76 | - | 58 | -40.7 | 83.47 | 0.00 | 0.39 | -0.17 | - |
| 2. Maximum Size | 95 | -56.4 | 117.08 | 1.30 | 2.79e-1 | -0.82 + 1.34 | - | 57 | -40.4 | 85.22 | 1.50 | 0.18 | -0.17 + 0.38 | - |
| 3. Median Depth | 95 | -57.4 | 118.91 | 3.13 | 1.12e-2 | -0.83 + -1.29 | - | 57 | -40.5 | 85.32 | 1.60 | 0.17 | -0.17 + -0.34 | - |
| 4. Max Size + Med Depth | 94 | -54.8 | 116.02 | 0.00 | 5.35e-1 | -0.87 + 1.09 +  -0.95 | - | 56 | -40.3 | 87.28 | 3.15 | 0.08 | -0.17 + 0.36 +  -0.32 | - |
| 1. **Accounting for taxonomic family as a fixed effect (GLM)** | | | | | | | | | | | | | | |
| 5. Family | 69 | -38.7 | 180.22 | 17.71 | 7.64e-5 | - | - | 34 | -20.8 | 168.96 | 8.32 | 0.01 | - | - |
| 6. Maximum Size + Family | 68 | -34.5 | 178.01 | 11.36 | 1.82e-3 | - | - | 33 | -20.7 | 179.60 | 10.11 | 0.002 | - | - |
| 7. Median Depth + Family | 68 | -37.1 | 183.31 | 16.66 | 1.29e-4 | - | - | 33 | -20.8 | 179.70 | 10.21 | NA | - | - |
| 8. Max Size + Med Depth + Family | 67 | -33.6 | 182.71 | 11.69 | 1.55e-3 | - | - | 32 | -20.7 | 191.20 | 12.03 | NA | - | - |
| 1. **Accounting for taxonomic family as a random effect (GLMM)** | | | | | | | | | | | | | | |
| 9. (1\|Family) | 95 | -60.4 | 124.76 | 9.13 | 5.57e-3 | -0.72 | 0.44 / 0.67 | 57 | -40.6 | 85.31 | 1.82 | 0.15 | -0.16 | 0.32 / 0.57 |
| 10. Maximum Size + (1\|Family) | 94 | -56.4 | 119.12 | 3.30 | NA | -0.82 + 1.34 | 0 / 0 | 56 | -40.4 | 87.06 | 3.36 | NA | -0.18 + 0.40 | 0.30 / 0.55 |
| 11. Median Depth + (1\|Family) | 94 | -57.1 | 120.32 | 4.53 | 5.54e-2 | -0.80 + -1.37 | 0.31 / 0.56 | 56 | -40.4 | 87.06 | 3.36 | NA | -0.15 + -0.40 | 0.37 / 0.61 |
| 12. Max Size + Med Depth + (1\|Family) | 93 | -54.8 | 118.07 | 2.00 | NA | -0.87 + 1.09 +  -0.95 | 0 / 0 | 55 | -40.2 | 89.16 | 4.99 | 0.03 | -0.17 + 0.37 +  -0.36 | 0.33 / 0.58 |

**Supplementary Table S4 | Model selection excluding static-status Endangered and Critically Endangered species from 1980–2015.** Generalised Linear Models and Generalised Linear Mixed-Effects Models testing the effect of maximum body size (cm, total length/wing span) and median depth (m) on whether a shark or ray species’ IUCN status is worsening (1) or not (0) in the Northeast Atlantic (*n* = 100) and Mediterranean Sea (*n* = 53). Models exclude the outlier Basking Shark. (*K* = degrees of freedom, logLik = negative log likelihood, AIC_c_ = AIC corrected for small sample size, ΔAIC = model ranking [0 is best, anything <2 is not significantly different from best, bold], AIC wt = AIC weight, Coef est = coefficient estimates, Ran effect var = variance of random effect [only c] , SD = standard deviation). Grey text represents models that have the same negative log likelihood as less complex models, hence they are removed from the calculation of AIC weights.

| **Model** | **Northeast Atlantic** | | | | | | | **Mediterranean Sea** | | | | | | |
| --- | --- | --- | --- | --- | --- | --- | --- | --- | --- | --- | --- | --- | --- | --- |
| **Response:**  Status Change  **Fixed effects:** | ***K*** | **logLik** | **AIC_c_** | **ΔAIC** | **AIC wt** | **Coef est** | **Ran effect var / SD** | ***K*** | **logLik** | **AIC_c_** | **ΔAIC** | **AIC wt** | **Coef est** | **Ran effect var** |
| 1. **Without accounting for taxonomic family (GLM)** | | | | | | | | | | | | | | |
| 1. Intercept | 101 | -70.0 | 142.05 | 45.41 | 1.37e-10 | -0.24 | - | 52 | -30.6 | 63.32 | 10.05 | 4.17e-3 | 1.02 | - |
| 2. Maximum Size | 100 | -51.9 | 107.93 | 11.15 | 3.77e-3 | -0.24 + 3.20 | - | 51 | -25.2 | 54.79 | 1.23 | 3.42e-1 | 1.38 + 2.61 | - |
| 3. Median Depth | 100 | -57.3 | 118.72 | 21.94 | 1.71e-5 | -0.38 + -2.52 | - | 51 | -29.3 | 62.91 | 9.36 | 5.87e-3 | 1.07 + -0.98 | - |
| 4. Max Size + Med Depth | 99 | -45.3 | 97.00 | 0.00 | 9.96e-1 | -0.33 + 2.87 +  -2.00 | - | 50 | -23.6 | 54.01 | 0.00 | 6.33e-1 | 1.47 + 2.81 +  -1.21 | - |
| 1. **Accounting for taxonomic family as a fixed effect (GLM)** | | | | | | | | | | | | | | |
| 5. Family | 76 | -39.0 | 181.52 | 33.39 | 5.59e-8 | - | - | 33 | -15.9 | 166.24 | 18.64 | 5.67e-5 | - | - |
| 6. Maximum Size + Family | 75 | -32.7 | 175.12 | 22.75 | 1.14e-5 | - | - | 32 | -15.9 | 179.79 | 20.63 | NA | - | - |
| 7. Median Depth + Family | 75 | -36.2 | 182.17 | 29.79 | 3.38e-7 | - | - | 32 | -14.2 | 176.48 | 17.32 | 1.10e-4 | - | - |
| 8. Max Size + Med Depth + Family | 74 | -29.9 | 176.12 | 19.26 | 6.56e-5 | - | - | 31 | -14.2 | 191.11 | 19.15 | NA | - | - |
| 1. **Accounting for taxonomic family as a random effect (GLMM)** | | | | | | | | | | | | | | |
| 9. (1\|Family) | 100 | -64.9 | 133.86 | 37.23 | 8.21e-9 | 0.27 | 2.40 / 1.55 | 51 | -29.6 | 63.32 | 10.07 | 4.12e-3 | 1.54 | 1.52 / 1.24 |
| 10. Maximum Size + random(family) | 99 | -51.9 | 109.90 | 13.15 | NA | -0.24 + 3.20 | 0 / 0 | 50 | -25.2 | 56.8 | 3.23 | NA | 1.38 + 2.61 | 7.9e-16 / 2.8e-8 |
| 11. Median Depth + random(family) | 99 | -55.3 | 116.80 | 20.05 | 4.41e-5 | -0.12 + -2.77 | 0.73 / 0.85 | 50 | -27.6 | 61.7 | 8.14 | 1.08e-2 | 2.09 + -1.84 | 2.92 / 1.71 |
| 12. Max Size + Med Depth + random(family) | 98 | -45.3 | 99.03 | 2.00 | NA | -0.33 + 2.87 +  -2.00 | 0 / 0 | 49 | -23.6 | 56.06 | 2.00 | NA | 1.47 + 2.81 +  -1.21 | 0 / 0 |

**Supplementary Table S5 | Model outputs for 2015 static IUCN status.** Generalised Linear Models testing the effect of maximum body size (cm, total length/wing span) and median depth (m) on whether a shark or ray species is threatened (1) or not (0) in the Northeast Atlantic and Mediterranean Sea. Models exclude the outlier Basking Shark. This was the top model for the Northeast Atlantic, whereas the top model for the Mediterranean Sea included body size only.

| **Model**  **Response:** IUCN status | **Northeast Atlantic** | | | | **Mediterranean Sea** | | | |
| --- | --- | --- | --- | --- | --- | --- | --- | --- |
| **Fixed effects:** | **Coefficient estimates** | **Standard error** | **Z-score** | ***K*** | **Coefficient estimates** | **Standard error** | **Z-score** | ***K*** |
| Intercept | -0.6544 | 0.2749 | -2.381 | 115 | 1.2439 | 0.4211 | 2.954 | 68 |
| Maximum size | 3.1653 | 0.6990 | 4.528 |  | 4.6445 | 1.1860 | 3.916 |  |
| Median depth | -2.0211 | 0.6847 | -2.952 |  | -2.4035 | 0.9325 | -2.578 |  |

**Supplementary** **Figure S1 | Effect of body size and depth distribution on IUCN status in Northeast Atlantic and Mediterranean sharks and rays.** Standardized effect sizes with standard error (95% confidence interval). Generalised Linear Models for the Northeast Atlantic (circles) and Mediterranean Sea (squares) showing the influence of maximum body size (cm, total length for sharks, skates, chimaeras or wing span for rays) and median depth (m) (fixed effects) on threat status (non-threatened = 0, threatened = 1; light colours) and on changing status between 1980 and 2015 (no change = 0, worsening status = 1; mid colours). Both models include all species except the outlier Basking Shark, *Cetorhinus maximus* (Northeast Atlantic *n* = 118, Mediterranean *n* = 71). Dark colours represent the same model for changing status, but with all “static-status” Endangered and Critically Endangered species excluded (i.e. those that did not change from the highest threat statuses since 1980; Northeast Atlantic *n* = 100, Mediterranean *n* = 53). IUCN listings include predicted-for Data Deficient species categorisations^25^. Data for maximum size and median depth were centred and standardised by two standard deviations. See Supplementary Table S1, S4 for model outputs.

**Supplementary** **Figure S2 | Various effects on the Red List Indices from removing potentially influential species groups.** Regional Red List Indices for Northeast Atlantic Ocean and Mediterranean Sea sharks and rays (a) including all regional species listings (Northeast Atlantic: *n*=119, Mediterranean Sea: *n*=72); (b) excluding boundary species listings (i.e. species with only the northerly-most edge of their geographic range within the assessment area; Northeast Atlantic: *n*=116, Mediterranean Sea: *n*=68); (c) excluding Data Deficient IUCN listings (Northeast Atlantic: *n*=98, Mediterranean Sea: *n*=60); and (d) excluding all migratory species as listed in Appendices I and II of the Convention on Migratory Species (except for Angel Shark *Squatina squatina* because it is endemic to the region; Northeast Atlantic: *n*=99, Mediterranean Sea: *n*=54). All examples excluding (c) include predicted-for categorisations for all Data Deficient species^1^. The Red List Index works on a scale of 0–1, where 0 indicates an entirely Extinct group and 1 represents a group listed entirely as Least Concern.

**Supplementary Figure S3 | Model diagnostics for Northeast Atlantic Generalised Linear Model plotted in Figure 4c.**

**Supplementary Figure S4 | Model diagnostics of standard residuals for Northeast Atlantic Generalised Linear Model plotted in Figure 4c.**

**Supplementary Figure S5 | Model diagnostics of outliers for Northeast Atlantic Generalised Linear Model plotted in Figure 4c.**

**Supplementary** **References**

1. Walls, R. H. L. & Dulvy, N. K. Eliminating the dark matter of Data Deficiency by predicting the conservation status of Northeast Atlantic and Mediterranean Sea sharks and rays. *Biol. Conserv.* **246**, 1–14 (2020).
